# Mobile Applications for Resting Tremor Assessment in Parkinson’s Disease: A Systematic Review

**DOI:** 10.3390/jcm12062334

**Published:** 2023-03-16

**Authors:** Paloma Moreta-de-Esteban, Patricia Martín-Casas, Rosa María Ortiz-Gutiérrez, Sofía Straudi, Roberto Cano-de-la-Cuerda

**Affiliations:** 1 Radiology, Rehabilitation and Physiotherapy Department, Nursing, Physiotherapy and Podiatry Faculty, Complutense of Madrid University, Plaza Ramón y Cajal 3, 28040 Madrid, Spain; 2 Department of Neuroscience and Rehabilitation, University of Ferrara, Via Luigi Borsari 46, 44121 Ferrara, Italy; 3 Department of Physiotherapy, Occupational Therapy, Rehabilitation and Physical Medicine, Health Science Faculty, Rey Juan Carlos University, Avda. Atenas S/N, 28922 Alcorcón, Spain

**Keywords:** assessment, mobile applications, Parkinson’s disease, tremor

## Abstract

(1) Background: Resting tremor is a motor manifestation present in most Parkinson’s disease (PD) patients. For its assessment, several scales have been created, but mobile applications could help in objectively assessing resting tremor in PD patients in person and/or remotely in a more ecological scenario. (2) Methods: a systematic review following the PRISMA recommendations was conducted in scientific databases (PubMed, Medline, Science Direct, Academic Search Premier, and Web of Science) and in the main mobile application markets (Google Play, iOS App Store, and Windows Store) to determine the applications available for the assessment of resting tremor in patients with PD using only the measurement components of the phone itself (accelerometers and gyroscopes). (3) Results: 14 articles that used mobile apps to assess resting tremor in PD were included, and 13 apps were identified in the mobile application markets for the same purpose. The risk of bias and of applicability concerns of the articles analyzed was low. Mobile applications found in the app markets met an average of 85.09% of the recommendations for the development of medical mobile applications. (4) Conclusions: the use of mobile applications for the evaluation of resting tremor in PD patients has great potential, but validation studies for this purpose are scarce.

## 1. Introduction

Parkinson’s disease (PD) is a neurodegenerative pathology characterized by a chronic, progressive, and irreversible evolutionary course in which there is a selective degeneration of the dopaminergic neurons of the substantia nigra of the midbrain [1,2]. This fact results in the four characteristic motor manifestations of the disease: bradykinesia, resting tremor, muscle rigidity, and postural instability [3]. Moreover, PD also exhibits non-motor alterations such as digestive disorders, sleep disturbances, and behavioral and cognitive changes that can precede motor symptoms for years [1,3].

Among the movement disorders present in PD is resting tremor, defined as an involuntary, rhythmic, and oscillatory movement of a body segment in the absence of movement [4,5]. It is usually an initial manifestation in 50% of cases, and it is present in 70–85% of all patients with PD [5]. Resting tremor is usually found in the distal area of the extremities and has a frequency between 3 and 6 Hz [5]. To assess tremor, there are several scales, although most of those available focus on intentional tremor [6]. Among those that focus on resting tremor, the Fahn–Tolosa–Marín Tremor Scale scores the tremor’s location, severity, and functional disability produced by it as being the most used both in the clinic and in research [7]. The Bain and Findley Clinical Tremor Scale scores the severity of tremors from 1 to 10 in different areas of the body [6]. Likewise, within section III of the Unified Parkinson’s Disease Rating Scale (UPDRS) there are items for resting tremor that asses the amplitude (measured in centimeters, with a tremor above 3 cm being severe) and the total time that tremor appears during the examination (measured as the percentage of time that it appears, with a tremor considered severe if it appears above 75% of the time of the examination) [8]. However, all these scales have a subjective component of assessment; there is a lack of objective tools that help to identify the worsening of a resting tremor, as well as to be able to weigh the effects of pharmacological and/or rehabilitation treatment in PD.

In the last years, the development of information and digital technology has allowed a change in the health paradigm and a new way of relating to health [9,10]. Among these advances, mHealth (information and communication technologies using mobile phones and/or tablets for medical purposes) stands out for both patients and professionals [11]. For patients with PD, the use of mobile applications has allowed them to immediately and inexpensively access a community of people with whom to interact, to obtain more information about their disease, to receive help to remember to take their medication, as well as access to other tools that facilitate their daily activities. For professionals, the use of mobile applications can allow a more agile and objective assessment of certain parameters, the planning and recommendation of treatment, and the follow-up of patients with PD quickly and accurately, overcoming the barriers of distance and time [12,13].

Specifically, the presence of tremor in the different stages of PD is a prognostic factor of functional impairment [14]. Therefore, having an easily applicable monitoring evaluation such as an app could facilitate the therapeutic needs of the patient in the evolutionary process of the disease. Consequently, it could be assumed that the use of mobile applications could allow an objective assessment of resting tremor in PD.

To our knowledge, two previous systematic reviews have been published regarding the use of mobile applications in PD [12,15]. Both studied the use of apps aimed at both treatment and assessment globally in PD. The present review focused on articles in scientific databases and on mobile applications available in the app market at the time. Another main difference between the previous systematic reviews and the present work is that our research focused on addressing the use of mobile applications using only the measurement components of the phone itself (a phone´s accelerometer and gyroscope) in patients with PD. In contrast to previous studies, we only included studies in which resting tremor was measured without voluntary activity of the patient (we did not include assessments through Archimedes’ spiral). Finally, we also analyzed the studies from the point of view of diagnostic reliability and application according to the recommendations for the development of medical mobile applications.

Therefore, the purpose of this work was to analyze the available evidence on mobile applications as well as the mobile apps available in the app markets, using only the measurement components of the phone itself, for the assessment of resting tremor in patients with PD.

## 2. Materials and Methods

### 2.1. Design

A systematic review was carried out following the recommendations of the PRISMA statement (Preferred Reporting Items for Systematic reviews and Meta-Analyses) for the development of systematic reviews and meta-analyses [16].

### 2.2. Search Strategy

An information search was conducted in electronic databases and other sources of information specific to the field of mobile applications (app markets) following the methodology of previous work on this subject [12,13].

Two independent reviewers carried out bibliographic searches in the following databases: PubMed, Medline, Science Direct, Academic Search Premier, and Web of Science, as well as a cross-search through references found in articles from these sources. In the case of a discrepancy, it was resolved by a third independent investigator.

The search strategy used in the databases and the search filters are detailed in Table 1.

### 2.3. Eligibility Criteria

Articles published between 2011 and 2021 in Spanish and English were considered. The lower limit of 2011 was chosen since the first reference in relation to the development of a mobile application in PD was described in that year [12].

The criteria for inclusion of articles for the systematic review were: (a) Patients diagnosed with idiopathic PD according to the criteria of the Brain Bank [17]. (b) Original studies published in electronic databases with patients, regardless of their methodological design. (c) Apps that used the measurement components of the phone itself (accelerometers and gyroscopes). (d) Assessment of resting tremor. (e) Articles in English or Spanish.

In parallel to the search in biomedical databases, a search was carried out for mobile applications related to resting tremor in PD in the main markets of mobile applications of Android (Google Play), iOS (App Store), and Windows Phone (Windows Store) systems.

In the absence of a strict protocol for searching mobile applications, it was conducted following the methodology of a previous systematic review on mobile applications in neurorehabilitation [13]. In the first phase, applications related to resting tremor from the bibliographic search were included. Subsequently, applications from the app markets were added. From all the apps, data were collected on the name, the operating system, the price, the type of user the app addressed, its logo, and a brief description of use.

### 2.4. Extracting Information and Managing Data

The following data were extracted: number and characteristics of participants; measurement protocol; outcome results; name and main features of the apps such as operating system; type of user; and a brief description.

### 2.5. Assessing the Quality of Evidence

To evaluate the methodological quality of the included articles, the QUADAS-2 scale was administered for a diagnostic test. This scale determines the risk of bias and clinical applicability of a study test, assigning a high, low, or unclear probability of risk of bias across four domains. As a result, a table and graph are presented in which the risk of bias and the applicability of the scale are visualized [18].

To quantify the level of risk of bias, a high, low, or unclear probability is assigned to four domains of the scale (patient selection, index test, baseline test, and flow times). This score is guided by questions to help judge the risk of bias dichotomously (affirmative/negative).

Similarly, for the clinical applicability of the tool, the domains of patient selection, index test, and reference test are scored. In this section of the scale, there are no guiding questions, but a question is always formulated to be solved. In the case of applicability in patient selection, it would be questioned whether there is a concern that the application of the diagnostic test being evaluated did not match the review topic.

To evaluate the quality of mobile applications found in the app markets, a critical analysis was carried out based on the “Recommendations for the design, use and evaluation of health apps” published by the “Junta de Andalucía” (Spain) in 2012. Aspects of design and relevance, quality and security of information, provision of services, and confidentiality and privacy were evaluated. All applications included in this review were assessed according to these quality criteria [19].

## 3. Results

**Figure 1:**
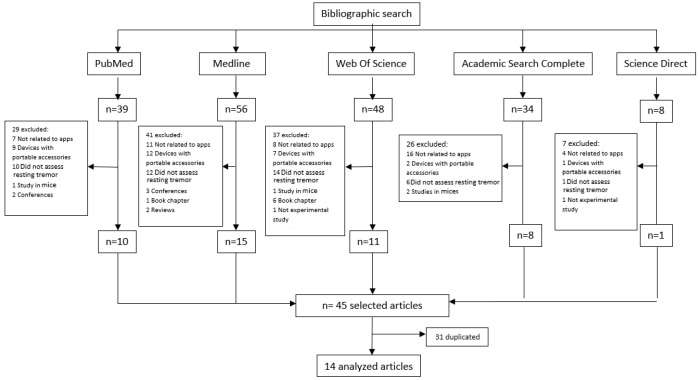
Flowchart of the bibliographic search.

A total of 185 articles were screened that were identified in the databases consulted. After removing duplicates and screening by title and abstract based on eligibility criteria, a total of 14 articles were included [20,21,22,23,24,25,26,27,28,29,30,31,32,33]. The screening process is presented in the flowchart in Figure 1.

### 3.1. Participants

A total of 510 individuals participated in the 14 studies included in this systematic review: 287 patients diagnosed with PD, 53 diagnosed with essential tremor, 1 diagnosed with Holmes Tremor, 7 undiagnosed, 12 patients that showed an undiagnosed tremor, 15 patients with dopamine transport deficit, 123 healthy participants, and 2 subjects without tremor.

### 3.2. Apps in Databases

Fourteen studies were analyzed in which nine mobile applications were presented to detect resting tremor in people with PD. The main characteristics of the articles included in the systematic review are presented in Table 2.

The methodological design of all articles found was observational: nine were specificity/sensitivity articles, two were diagnostic concordance studies [20,22], and three were descriptive observational [24,29,31].

Ten articles used mobile applications marketed for or pending commercialization [20,21,22,23,25,26,29,30,31,32]. Only Chen et al. [23] in 2020 used the same mobile application that Lipsmeier et al. [30] used in 2018 in their study to create a sophisticated and accurate computer model, with a study design very similar to that of Lipsmeier et al. [30]. The remaining articles were based on applications exclusively designed for such scientific studies [24,27,28,33].

Two of the articles included in our systematic review described in detail the computer process for obtaining the data [23,24]. Chronowski et al. [24] presented the computer model used to analyze the data of the application, concluding that despite the good accuracy it could not yet be used as a diagnostic tool because it showed 85% diagnostic accuracy, which the authors considered insufficient. Chen et al. [23] used data from two previous experiments in which participants carried a smartphone in a special case that was attached to the upper limb to collect data of resting tremor with the application “Roche PD Mobile Application v1”. Then, they designed the computer model to analyze this data, concluding that the most significant variables to discriminate between patients with PD and healthy participants were resting tremor and dexterity.

Despite the importance of medication in the control of resting tremor, nine of the articles did not detail whether they performed the measurements in periods “on” or “off” medication [20,22,23,24,25,26,29,30,31,32,33]. Both Kostikis et al. [28] and Barrantes et al. [21] performed the measurements in periods “on” medication and only Kassavetis et al. [27] did so in the “off” period of the medication.

Only two authors compared mobile apps with another objective tool for detecting resting tremor [20,22]. Araujo et al. [20] compared an app with needle electromyography in key upper limb muscles, and Brummelen et al. [22] compared the results of two mobile applications with an accelerometer under laboratory conditions. In both articles, a good correlation was found between the measurements of the mobile applications and the indicated diagnostic tools.

In seven of the articles, the aim was to determine the presence of PD compared to healthy participants with the detection of resting tremor by a mobile application [21,23,25,26,28,30]. Barrantes et al. [21] and Woods et al. [33] proposed mobile applications to differentiate between PD and essential tremor according to the difference in amplitude between these two types of tremor.

Although most articles used the UPDRS scale to measure the initial health status in patients, only four articles additionally correlated the results obtained through the mobile applications and the items intended for the assessment of tremor in the UPDRS (section III). In all of them, a significant correlation was found for resting tremor [23,27,29,30].

In relation to the resting tremor assessment protocol, six authors evaluated it by tying the mobile phone to the patient’s hand for 30 s in a relaxed position [20,21,24,25,28,32]. The rest of the authors evaluated it by letting the tool rest on the palm of the hand [26,28,29], actively holding it in the hand [22,23,33], or using the smartphone but without specifying how [26,31]. Only three articles recorded daytime tremor, including activities of daily living (ADLs), with good results in distinguishing between healthy participants and patients with resting tremor [23,26,30].

### 3.3. App Markets

**Figure 2:**
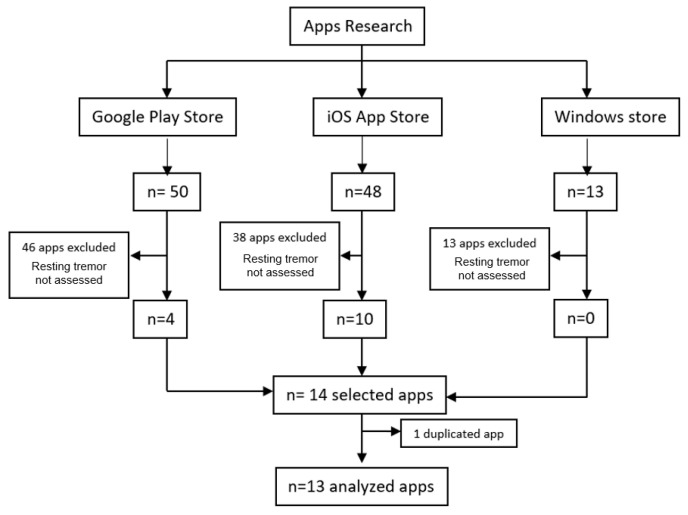
Flowchart of the search for mobile applications in app markets.

In the search carried out in the main mobile application markets (Google Play Store, iOS App Store, and Windows App Store), 13 apps available for the evaluation of resting tremor in PD were selected, of which 4 were available in the Google Play Store and 9 in the iOS App Store, while 1 was available on both platforms. Figure 2 shows the search process. Table 3 presents the operating system, the type of user, the cost, the language, and a brief description of use of each of the apps.

Of the 13 apps found, 9 were free and 4 were not; taking into account the type of user at whom they were aimed, 2 of them were intended for use by patients, 5 by professionals, and 6 by both professionals and patients.

Of the 13 applications analyzed, 7 were designed exclusively to measure tremor quantitatively, while the remaining 6 apps also allowed the evaluation of other symptoms such as balance, oral tremor, gait, bradykinesia, and cognitive symptoms. Of all the articles analyzed, only the mobile application described by Araujo et al. [19] from the bibliographic search was available in the application markets, specifically for the iOS system, to measure tremor in amplitude, frequency, and power both by professionals and by the patients themselves.

### 3.4. Quality of Evidence

**Figure 3:**
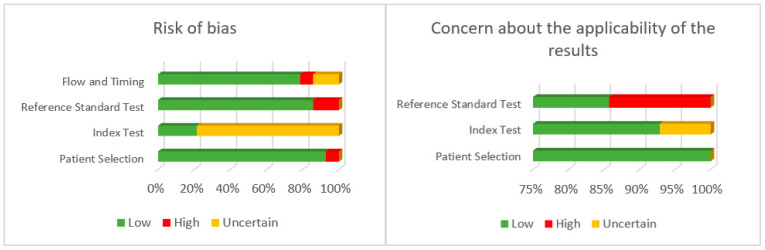
Risk of bias of the articles analyzed.

The results regarding the methodological quality of the articles and their risk of bias are detailed in Figure 3 and Table 4. Overall, the risk of bias presented in the included articles was low. However, regarding the index test (the diagnostic tool that was evaluated), it should be noted that in most cases there was an uncertain risk of bias. This is due to the lack of information in the articles about the threshold to determine the positivity or negativity of the test. The lack of information on whether the authors knew the results of the reference test before knowing the results of the index test is also remarkable, and that is considered to increase the chances of bias.

The quality of the apps found was measured by “The recommendations of the Junta de Andalucía for the design of health applications” [19]. Table 5 shows the main characteristics of the apps in terms of design and relevance, the quality and security of information, the provision of services, and confidentiality and privacy. The average percentage of compliance of the included apps, according to the criteria described, was 85.09%. 

## 4. Discussion

To achieve the objective of this review, a total of 14 articles [19,20,21,22,23,24,25,26,27,28,29,30,31,32] were analyzed in which 9 mobile applications were presented. Further, 13 applications available in the app markets were identified.

Six articles used tremor evaluation through apps as a diagnostic tool in people with PD, people with essential tremor, and healthy participants [21,23,24,26,28,30]. However, none of these studies aimed to validate the app as a diagnostic instrument for tremor but only as a screening tool among people with or without the presence of tremor. In this sense, only two studies compared the measurements made through mobile applications (“LiftPulse”, “iSeismometer”, “StudyMyTremor”, “Make Helsinki app”, “Centre for Human Drug Research app”) with an objective validation tool, namely, needle electromyography and an accelerometer in laboratory conditions, finding a good correlation between both measurements [20,22]. Nevertheless, of the five applications studied by these authors, only “StudyMyTremor” is currently available on the market.

To determine the suitability of the apps as instruments to monitor the health status of people with PD, the concordance of two apps (“Roche PD Mobile Application v1”and “Sentient Tracking of Parkinson’s”) with UPDRS was studied [23,27,29,30]. The authors used this scale as a reference measure because it is the most used in the clinical field even though other scales such as the Fahn–Tolosa–Marín Scale have shown to be more sensitive to changes in tremor associated with exogenous factors such as the use of medication [34,35]. These apps showed a good correlation with UPDRS when both measuring instruments were applied in the same way for tremor recording. Only in the study by Woods et al. [33], tasks evaluated through the app were modified regarding those proposed in the UPDRS scale, including distraction tasks, and with different focuses of attention. Data provided by both instruments showed that resting tremor behaved differently when the patient was asked to perform tasks involving cognitive processes, with the results differing in patients with PD and ET.

Although most of the authors proposed a very similar protocol regarding the assessment of tremor (patient seated with their arms along their body in a relaxed position), variability was observed in terms of the smartphone location. Some authors tied the smartphone to the patient’s hand allowing it to be relaxed [20,21,24,25,28,32], while others asked patients to actively grasp the smartphone [21,22,32] or hold it on the palm of their hand with the forearm in supination [27,29,30]. This lack of homogeneity in the assessment generates doubts about the results of these measurements, since only patients who were assessed with the smartphone tied to their hand would be truly at rest. Moreover, in our systematic review, 9 of the 14 articles did not specify whether patients were in on/off periods of medication [20,22,24,25,26,30,31,32,33]. The data need to be taken with caution, since the absence of information on the methodology of the included studies makes comparison between them difficult.

Two previous systematic reviews about the use of apps in PD were identified [12,15]. One of them studied apps available in different operating systems (iOS and Android) [15]. The other review classified the types of apps and their usefulness for assessment and/or treatment in PD [12]. However, the aim of the current review was to analyze the use of apps only in resting tremor in PD patients. The review by Linares-del Rey et al. [12] included five articles on the measurement of resting tremor and eight apps in the app markets. In this review, three of these articles were included, and the remaining two were excluded because they were conferences [25,28,32]. In the review by Estévez-Martín et al. [15] that only analyzed the app markets, 25 applications for measuring tremor at rest were found. This difference in the applications found between the present review and the ones carried out by Linares-del Rey et al. [12] and Estévez-Martín et al. [15] is because the present study did not include applications that measured tremor during a specific task, such as writing or drawing (Archimedes’ spiral), to assess resting tremor. Another main difference between the previous systematic reviews and the present one is that our research was focused on addressing the use of mobile applications using only the measurement components of the phone itself (the phone´s accelerometer and gyroscope) in patients with PD.

The methodological quality of the articles included in this review showed a low risk of bias. However, the risk of bias in the index test (the one being validated) was mostly uncertain. This was because in most articles it was unknown whether the authors knew the result of the reference test before performing the validation of their tool. It was also unknown how much time elapsed between the two assessments, which also increases the risk of bias. Regarding the applicability, a low risk was found except for the reference test, since many articles do not correlate the score obtained through mobile applications with the UPDRS. To our knowledge, there are no previous reviews that have studied the risk of bias from research on apps in the assessment of resting tremor in PD. However, in the review conducted by Linares del Rey et al. [12] the JADAD scale was included to assess the methodological quality of the studies found, regardless of their aim, although this scale could only be administered to 17 of the 26 articles due to the type of study (research protocols). Having used different tools to assess the methodological quality, it is not possible to make a comparison between the articles included in both reviews. Therefore, in the present review we decided to use the QUADAS-2 scale to assess the quality of the studies, since they were observational studies, and this scale is specifically designed to assess the quality of diagnostic tools included in a systematic review.

As for the applications found in the app markets, 13 available apps were identified, in contrast to the review by Linares del Rey et al. [12] in which, of the 8 apps found that focused on the assessment of resting tremor, only 2 (“StudyMyTremor” and “Tremor12”) remain on the market. Moreover, 5 of the 23 applications in the review by Estévez-Martín et al. [15] remain on the market (“cloudUPDRS”, “StudyMyTremor”, “Tremor12”, “Tremor Measurer”, and “Parkinson’s Lifekit”). This indicates that the market for mobile applications as an instrument for measuring health status is volatile. This lack of maintenance of the apps on the servers also makes it difficult to transfer the scientific evidence to clinical use.

Most authors highlight a lack of quality standards that allow users to determine the most suitable apps for their choice [26,36,37]. That is why, in this systematic review, we relied on the recommendations of the Junta de Andalucía of 2012 for the design, use, and evaluation of health apps [19]. There are other quality criteria such as those of Belloch-Ortí [38] created for the choice of multimedia resources in terms of qualitative criteria of general description, typology, requirements, technical characteristics, and aesthetic aspects. However, the criteria of the Junta de Andalucía were chosen since they are specifically designed for mobile applications in health. In general, the apps fulfilled their function and were easy to handle; only two apps (“Tremor Measurer” and “Tremor Measurer lite”) from the same developer were considered unintuitive and did not present clear guidelines. In addition, there were six apps (“ParkinsonAI”, “Tremor Measurement”, “Patana AI”, “StudyMyTremor”, “Tremor Measurer Lite”, and “TREMOR12”) whose developers had not shared the privacy policy with the sales platform, although the user can request it after downloading. This finding indicates a high risk in terms of data protection. Finally, there is only one application (“Patana AI”) that lacks revisions or updates, so its use may be discouraged due to the lack of review of the contents. It should be noted that most of the apps found would not have been validated in their a clinical context, nor approved by health agencies as proposed by Meulendijk et al. [39]. The only application found in the app market that was also found in the literature search in the study by Araujo et al. [20] was “StudyMyTremor”.

The results of this systematic review highlight the gap between research and commercial fields. While the papers analyzed include different methodologies for the design and evaluation of mobile applications, the available applications for assessing resting tremor in patients with PD have great variability in their quality characteristics and their validation process (if they exist). For this reason, future research should consider our findings to improve the relationship between the development of a mobile application and its validation in order to provide reliable and valid instruments to patients and health professionals. Firstly, research is needed to validate an app as a diagnostic instrument for resting tremor in patients with PD, comparing its results with objective instruments such as electromyography, accelerometers, inertial sensors, etc. Secondly, a comparison between the results obtained by an app and sensitive clinical scales such as the Fahn–Tolosa–Marín Scale and Bain and Findley Clinical Tremor Scale is needed due to their wide and easy use in clinical contexts. Thirdly, the protocol of evaluation should be better stablished because although most of the authors proposed a protocol with the patient seated with their arms along their body in a relaxed position, the smartphone location must be better defined; the smartphone should be located and tied to the patient’s hand, allowing it to be relaxed since resting tremor is present when no action is required. Fourthly, if medication effects are being tested by an app, data must be recorded in “on” and “off” periods of medication to have a complete characterization of resting tremor in patients with PD. However, if data are being taken as rehabilitation results, the results should be mainly taken in the “on” phase of the medication cycle, as this is the period during which patients perform most of their activities of daily living. Fifthly, the few applications that have proven to be valid and reliable are not available on the market except two (“StudyMyTremor” and “Tremor12”), indicating that app markets are too volatile and separated from the research field. Sixthly, the apps used should follow quality standards to determine their choice, paying special attention to aspects related to data protection, possible updates, and usability. Future studies must consider patients’ perceptions about using apps for resting tremor assessment since none of the mobile applications found in the app markets passed this examination; moreover, in the study by Motolese et al. 2020 [31] not all the participants were satisfied with the app when asked about it. So, future studies should address this issue. Given all this, it is necessary that scientists and the commercial industry make an effort to validate the apps available on the market or favor the development of apps that demonstrate their reliability and validity prior to making them available to patients and professionals to minimize errors and associated risks.

The present systematic review has several limitations. Firstly, the uncertain risk shown by the QUADAS-2 scale in some items calls for caution when recommending the use of certain apps in the assessment of resting tremor in PD. Secondly, due to the constant change in the application markets and their updates, some applications present in this review may not be available in the future or could be used with other devices such as tablets or smartwatches. Finally, the limitation of the language of the articles included (English and Spanish), as well as those derived from the inclusion criteria, could have meant that not all mobile applications of interest were included at the time of the search.

## 5. Conclusions

The present systematic review identified 14 articles that used mobile apps to assess resting tremor in PD patients. In addition, 13 apps were identified in the mobile application markets for the same specific purpose. The risk of bias and the risk of applicability concern of the articles analyzed were low. The mobile applications found in the app markets met an average of 85.09% of the recommendations for the development of medical mobile applications.

Mobile apps that are easy to manage and offer objective data could be beneficial for detecting and monitoring changes in resting tremor in PD patients. This could allow an easier and more precise monitorization of pharmacological and rehabilitation approaches for patients with PD. Therefore, the use of mobile applications for evaluating resting tremor in PD patients seems to have great potential. However, validation studies are necessary to recommend their use prior to their commercialization.

## Figures and Tables

**Table 1:** Database search strategy.

	Results
**PUBMED (Advance)—Search 1**	
#1 “Parkinson Disease” [Mesh]	76,971
#2 “Mobile Applications” [Mesh]	10,081
#3 “Tremor” [Mesh]	10,406
#4 #1 AND #2 AND #3	3
Filters: 2011–2021	3
**Total**	**3**
**PUBMED (Advance)—Search 2**	
#1 “Parkinson Disease” [TA]	13,991
#2 “Parkinson” [TA]	125,152
#3 #1 OR #2	125,152
#4 “Mobile Applications” [TA]	2739
#5 “App” [TA]	37,469
#6 “Smartphone” [TA]	17,921
#7 #4 OR #5 OR #6	51,979
#8 “Tremor” [TA]	22,518
#9 “Rest tremor” [TA]	556
#10 “Tremor Parkinson” [TA]	80
#11 #8 OR #9 OR #10	22,518
#12 #3 AND #7 AND #11	38
Filters: 2011–2021	38
**Total**	**38**
**MEDLINE/EBSCO (Advance)**	
S1 “Parkinson” [AB]	1,077,993
S2 “Tremor” [AB]	22,569
S3 “Mobile Applications” [AB]	1582
S4 “App” [AB]	36,617
S5 “Smartphone” [AB]	18,495
S6 S3 OR S4 OR S5	50,754
S7 S1 AND S2 AND S6	56
Filters: 2011–2021	56
**Total**	**56**
**WEB OF SCIENCE (Advance)**	
#1 Smartphone [Topic]	42,768
#2 Mobile application [Topic]	129,396
#3 App [Topic]	62,309
#4 Parkinson [Topic]	149,640
#5 Tremor [Topic]	31,585
**#6 #1OR #2 OR #3**	213,036
**#7 #6 AND #4 AND #5**	81
Filters: 2011–2021, Articles	48
**Total**	**48**
**ACADEMIC SEARCH PREMIER/EBSCO (Advance)**	
#1 Smartphone OR App OR Mobile applications	126,614
#2 Parkinson	90,117
#3 Tremor	40,406
#4 #1 AND #2 AND #3	35
Filters: 2011–2021	34
**Total**	**34**
**SCIENCE DIRECT (Advance)**	
#1 (“Smartphone” OR “App” OR “Mobile application”) AND “Parkinson” AND “Tremor” [Title, abstract or author-specified keywords]	8
Filters: 2011–2021	8
**Total**	**8**

**Table 2:** Characteristics of the studies included in the review.

Author and Year	Participants	Method	Results
Araújo et al., 2016 [20]	*n* = 22 (12 PD, 9 ET, 1 HT)	3 apps were tested by an iPhone (“**LiftPulse**”, “**iSeismometer**”, “**StudyMyTremor**”) tied to the patient’s hand, while needle EMG data were collected from the most relevant muscles in UL tremor.	All three apps showed good correlation with needle EMG, with statistically significant results. Although the results of all three were very similar, “LiftPulse” showed greater correlation.
Barrantes et al., 2017 [21]	*n* = 52 (17 PD, 16 ET, 7 undiagnosed, and 12 healthy participants)	Tremor data were collected through the “**SensoryLog**” app by tying the mobile phone to the hand for 30 s at rest and 30 s with the arms stretched at 90° of shoulder flexion while sitting.	Part 1. Differentiate patients with tremor from healthy patients with a specificity of 83.3% and a sensitivity of 97.96%. Part 2. Discriminate between patients with PD and ET: 27 patients out of 34 were correctly classified (84.38% accuracy).
Brummelen et al., 2020 [22]	*n* = 20 (10 PD and 10 ET)	Comparing the measurement of tremor simultaneously between a laboratory accelerometer with different equipment (iPhone, iPod, Apple watch^®^) with two apps: “**Make Helsinki app**” and “**Centre for Human Drug Research app**” by holding the mobile in the hand.	The tremor frequency peaks were similar between the laboratory accelerometer and the measuring equipment in both PD and ET. Greater amplitude of the tremor was recorded in the equipment that was placed more distally.
Chen et al., 2020 [23]	*n* = 72 (37 PD, 35 healthy participants)	Through data collected by a mobile app (“**Roche PD Mobile Application v1**”) such as gait, balance, dexterity, voice, and resting and postural tremor. It was intended to create a computer model to classify patients with or without PD and its severity. Patients had to perform activities with the mobile every day for 17 days; the data are collected when they hold the mobile in their hand.	They found that the most important characteristics to differentiate PD patients and healthy participants are resting tremor and dexterity. The model had an accuracy of 0.972, specificity of 0.971, and sensitivity of 0.973. Good correlation was found with the MDS-UPDRS scale. Greater relevance was found in the characteristics of dexterity, gait, and tremor at rest.
Chronowski et al., 2020 [24]	Healthy participants and PD patients. 49 samples were taken.	Thanks to an app on a smartphone tied to the patient’s hand, voice and resting tremor and intentional data were collected to discriminate between patients with PD and healthy participants.	85% accuracy was demonstrated by distinguishing patients with PD and healthy participants, but aspects of the interface need to be improved to make data analysis easier.
Fraiwan et al., 2016 [25]	*n* = 42 (21 PD, 21 healthy participants)	Data were collected through the “**Android Mobile**” **app** tied to the patient’s hand, which transmitted data collected from the smartphone’s accelerometer to a computer to be analyzed. Measurements were made for 30 s at rest to measure tremor.	The app and the data analysis system presented had 95% accuracy, 95% sensitivity, 95% specificity, with a kappa coefficient of 90%, diagnosing patients with PD due to resting tremor.
García-Magariño et al., 2016 [26]	Study 1, *n* = 21 PD (11 patients with tremor) Study 2, *n* = 3 PD (1 patient with tremor)	An app (“**Hand Trembling detector App**”) was designed to distinguish hand tremor in ADL. Study 1: The participants carried the smartphone with the app in their pocket and performed ADL. Study 2: Participants carried the smartphone for several hours at a time per day.	Study 1: The app was able to detect tremor with 95.83% sensitivity, 99.51% specificity, and an accuracy of 99.41%. Study 2: The app discriminated between tremor and normal movements in ADL, showing high specificity and sensitivity.
Kassavetis et al., 2015 [27]	*n* = 14 (patients with dopamine transport deficit)	Using an application on a smartphone, tremor (rest, postural, and action) and bradykinesia were measured to correlate them with MDS-UPDRS. The participants were measured in off-medication periods with the mobile phone on the palm of the hand in supination.	A significant correlation was found for resting and postural tremor as well as bradykinesia with the MDS-UPDRS scale, but not with action tremor (due in part to the characteristics of the sample).
Kostikis et al., 2015 [28]	*n* = 45 (25 PD and 20 healthy participants)	Thanks to an app also available in web version on a smartphone, resting and postural tremor in the hands of the participants were measured. The data were collected with the mobile phone tied to the patient’s hand.	82% of participants with PD and 90% of healthy participants were correctly identified. Better specificity and sensitivity were found in the data obtained from the gyroscope than from the app’s accelerometer.
Kuosmanen et al., 2020 [29]	*n* = 13 (13 PD)	The mobile “**Sentient Tracking of Parkinson’s**” (**STOP**) **app** measured the tremor through a game in which they held the phone on the palm of their hand for 13 s. The goal was to determine whether the app could detect and quantify tremor, as well as differences in tremor with and without medication.	The app was able to detect and quantify the severity of the tremor. A significant correlation was found with the items of the UPDRS III scale referring to tremor. No difference was found in tremor in patients with or without medication.
Lipsmeier et al., 2018 [30]	*n* = 79 (44 PD, 35 healthy participants)	Through data collected by a mobile app (“**Roche PD Mobile Application v1**”) of tremor at rest, bradykinesia, rigidity, postural instability, gait, and voice for 30 s and carrying the mobile all day. They performed two experiments, one lasting 6 months and the other 6 weeks. The data of resting tremor were collected with the mobile on the palm of the hand.	The app was able to discriminate PD patients from healthy participants with excellent reliability for tremor and moderate to good for the rest of the characteristics. It demonstrated moderate to good test–retest reliability. There was also a significant correlation with the MDS-UPDRS scale, except for the voice item. In addition, the app was able to discriminate other phenomena in Parkinson’s patients. Adherence was 61% in the long experiment and 100% in the short experiment.
Motolese et al., 2020 [31]	*n* = 54 PD	During the COVID-19 lockdown, patients had to use the “**EncephaLog HomeTM**” mobile application at least 2 times a week for 3 weeks to monitor symptoms (tremor among them).	83.3% of participants used the app at least once. 53.7% of the participants showed average conformity with the app, and 29.6% were very satisfied with it. Adherence was 38.7%. 18.5% underwent PD treatment changes upon request due to clinical reasons. All performed therapeutic interventions were routine modifications of ongoing medications. None was driven by the app outcomes, due to the observational nature of the study.
Pan et al., 2015 [32]	*n* = 40 PD	The mobile application “**PD Dr**” was developed to detect the motor symptoms of Parkinson’s (tremor and gait difficulties). Participants performed a test for 5 min to obtain the data, in which they carried the mobile tied to their hand.	Resting tremor obtained a sensitivity of 0.77 and an accuracy of 0.82. A strong correlation was demonstrated between test results and disease severity in participants.
Woods et al., 2014 [33]	*n* = 32 (14 PD and 18 ET)	Thanks to an app designed specifically for the study, they evaluated tremor at rest by holding the mobile phone with their hand, as well as during attention and distraction tasks to discriminate between patients with PD and ET.	92% of patients were well discriminated.

ADL: activity of daily living; PD: Parkinson’s disease; EMG: electromyography; MDS-UPDRS: Movement Disorder Society-Sponsored Revision of the Unified Parkinson’s Disease Rating Scale; TE: essential tremor; HT: Holmes tremor; UL: upper limb; UPDRS: Unified Parkinson’s Disease Rating Scale.

**Table 3:** Main features of apps for the assessment of resting tremor in PD.

Name	Logo	Operating System	Price	Users	Brief Description
cloudUPDRS	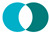	Google Play	Free	Professionals and patients participating in the study	App to measure gait, tremor, and reaction time. The test can be performed complete or in parts.
MyTremorApp	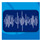	Google Play	Free	Professionals and patients	App to measure hand tremor and bradykinesia.
ParkinsonAI	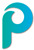	Google Play/iOS	Free	Patients	App to measure tremor and posture. It also allows you to record medical history and proposes exercises and diets for the control of symptoms.
Tremor Measurement	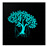	Google Play	EUR 1,39	Professionals	App to assess tremor in amplitude and frequency.
Cepha	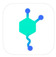	iOS	Free	Professionals and patients	App to measure dysphonia, resting tremor, action tremor, and postural tremor. It was created to distinguish essential tremor from PD in a study not cited by the developers.
CYPD	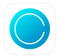	iOS	Free	Professionals and patients	App to monitor symptoms and medication effectiveness. This information is sent to the clinician. Can also be used with an Apple watch^®^.
Parkinson’s LifeKit	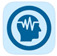	iOS	EUR 3.99	Patients	App to manage PD. Presents cognitive and motor tests (voice, tapping, and tremor); a personal diary; medication reminders; and a result graphic to see variations in symptoms.
Patana AI	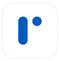	iOS	Free	Professionals	App to evaluate tremor, posture, and movement.
StudyMyTremor	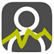	iOS	EUR 3.99	Professionals and patients	App to evaluate tremor in amplitude and frequency. You can record the data in a calendar to compare the data.
Tremor Analysis	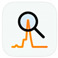	iOS	Free	Professionals and patients	App to assess tremor in frequency. It can be customized by choosing which parameters you want to measure. It can also be used with an Apple watch^®^.
Tremor Measurer	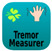	iOS	EUR 1.99	Professionals	App to assess tremor quantitatively.
Tremor Measurer Lite	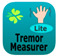	iOS	Free	Professionals	App to assess tremor quantitatively.
TREMOR12	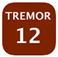	iOS	Free	Professionals	App to measure tremor parameters and analyze them later. Updated to also be used with Apple watch^®^.

**Table 4:** Risk of bias of selected articles.

	Risk of Bias				Concern about the Applicability of the Results		
Article (App)	Patient Selection	Index Test	Reference Standard Test	Flow and Timing	Patient Selection	Index Test	Reference Standard Test
Araújo et al., 2016 [20] (“**LiftPulse**”, “**iSeismometer**”, “**StudyMyTremor**”)	L	U	L	L	L	L	L
Barrantes et al., 2017 [21] (“**SensoryLog**”)	L	U	L	L	L	L	L
Brummelen et al., 2020 [22] (“**Make Helsinki app**”, “**Centre for Human Drug Research app**”)	L	U	L	L	L	L	L
Chen et al., 2020 [23] (“**Roche PD Mobile Application v1**”)	L	U	L	L	L	U	L
Chronowski et al., 2020 [24]	L	U	L	H	L	L	L
Fraiwan et al., 2016 [25] (“**Android Mobile**” **app**)	L	L	H	U	L	L	H
García-Magariño et al., 2016 [26] (“**Hand Trembling detector App**”)	H	U	H	U	L	L	H
Kassavetis et al., 2015 [27]	L	L	L	L	L	L	L
Kostikis et al., 2015 [28]	L	U	L	L	L	L	L
Kuosmanen et al., 2020 [29] (“**Sentient Tracking of Parkinson’s**” (**STOP**))	L	U	L	L	L	L	L
Lipsmeier et al., 2018 [30] (“**Roche PD Mobile Application v1**”)	L	L	L	L	L	L	L
Motolese et al., 2020 [31] (**EncephaLog HomeTM**)	L	U	L	L	L	L	L
Pan et al., 2015 [32] (“**PD Dr**”)	L	U	L	L	L	L	L
Woods et al., 2014 [33]	L	U	L	L	L	L	L

L: low risk; H: high risk; U: uncertain risk.

**Table 5:** Main quality characteristics of the apps analyzed.

App	Design and Relevance				Information Quality and Security						Provision of Services				Confidentiality and Privacy		Compliance of Recommendation (%)
	Relevance	Accessibility	Design	Usability/Testing	Audience Adequacy	Transparency	Authorship	Revisions	Contents and Sources of Information	Risk Management	Technical Support	E-Commerce	Bandwidth	Publicity	Privacy and Data Protection	Logical Security	
cloudUPDRS	✓	✓	✓	NE	✓	✓	✓	✓	✓	✓	✓	✕	✓	✓	✓	✓	87.5%
MyTremorApp	✓	✓	✓	NE	✓	✓	✓	✓	✓	✓	✓	✓	✓	✓	✓	✓	93.75%
ParkinsonAI	✓	✓	✓	NE	✓	✓	✓	✓	✕	✓	✓	✓	✓	✓	✓	✓	87.5%
Tremor Measurement	✓	✓	✓	NE	✓	✓	✓	✓	✕	✓	✓	✓	✓	✓	✓	✓	87.5%
Cepha	✓	✓	✓	NE	✓	✓	✓	✓	✓	✓	✓	✕	✓	✓	✓	✓	87.5%
CYPD	✓	✓	✓	NE	✓	✓	✓	✓	✕	✓	✓	✕	✓	✓	✓	✓	81.25
Parkinson’s LifeKit	✓	✓	✓	NE	✓	✓	✓	✓	✕	✓	✓	✓	✓	✓	✓	✓	87.5%
Patana AI	✕	✓	✓	NE	✓	✓	✓	✕	✓	✓	✓	✕	✓	✓	✓	✓	75%
StudyMyTremor	✓	✓	✓	NE	✓	✓	✓	✓	✓	✓	✓	✓	✓	✓	✓	✓	93.75%
Tremor Analysis	✓	✓	✓	NE	✓	✓	✓	✓	✕	✓	✓	✓	✓	✓	✓	✓	87.5%
Tremor Measurer	✕	✓	✓	NE	✓	✓	✓	✓	✕	✓	✓	✕	✓	✓	✓	✓	75%
Tremor Measurer Lite	✕	✓	✓	NE	✓	✓	✓	✓	✕	✓	✓	✕	✓	✓	✓	✓	75%
TREMOR12	✓	✓	✓	NE	✓	✓	✓	✓	✕	✓	✓	✓	✓	✓	✓	✓	87.5%

NE: not specified; ✕: does not meet the criteria; ✓: does meet the criteria.

## Data Availability

Not applicable.
